# Comparable rates of catheter-related bloodstream infections between non-tunneled and tunneled hemodialysis catheters: a retrospective single-center study

**DOI:** 10.1093/ckj/sfaf392

**Published:** 2025-12-16

**Authors:** Matej Zrimšek, Jakob Gubenšek

**Affiliations:** Department of Nephrology, University Medical Centre Ljubljana, Ljubljana, Slovenia; Faculty of Medicine, University of Ljubljana, Ljubljana, Slovenia; Department of Nephrology, University Medical Centre Ljubljana, Ljubljana, Slovenia; Faculty of Medicine, University of Ljubljana, Ljubljana, Slovenia

**Keywords:** catheter-related blood stream infection, chronic hemodialysis, hemodialysis catheter, pre-curved jugular non-tunneled catheters, tunneled hemodialysis catheters

## Abstract

**Background:**

A hemodialysis catheter may serve as a short- or medium-term vascular access solution. Current guidelines suggest restricting non-tunneled catheter use to 2 weeks, partially based on studies using straight non-tunneled jugular catheters, which have now been widely replaced with pre-curved catheters. We compared the rate of catheter-related blood stream infections (CRBSIs) and possible CRBSIs (PCRBSIs) of pre-curved non-tunneled and tunneled catheters in our hemodialysis center.

**Methods:**

This was a retrospective study including patients dialyzed on an outpatient basis between 1 January 2018 and 1 July 2024, with a follow-up until 1 March 2025. The primary aim was to compare the rates of CRBSIs.

**Results:**

In 301 patients, 625 non-tunneled single lumen catheter pairs and 53 double lumen tunneled catheters were used. There were 53 CRBSIs in non-tunneled and 10 in tunneled catheters, with identical incidence rate (0.48/1000 catheter-days in both groups). Analyzing CRBSIs and PCRBSIs together also showed similar infection rates [0.66 vs 0.58, incidence rate ratio (IRR) with 95% confidence interval 1.14 (0.6–2.1), *P* = .68]. Two subanalyses were made: CRBSI IRR in 27 patients with both types of catheters during study period was 1.37 (0.55–3.41, *P* = .49) and 2.01 (0.52–7.72, *P* = .47) in 36 patients after their first CRBSI. Time to CRBSI was also comparable in all analyses.

**Conclusions:**

Our study found no significant difference in the incidence of CRBSIs. We conclude that prolonged use of non-tunneled pre-curved catheters, which are easily managed, is a viable option for patients awaiting construction of arteriovenous fistula, insertion of a peritoneal catheter or kidney transplantation in a reasonable time. Promising results on long-term use from this study need to be confirmed in prospective studies.

KEY LEARNING POINTS **What was known:**

- Guidelines strongly recommend the use of tunneled cuffed hemodialysis catheters almost exclusively and suggest restricting the use of non-tunneled hemodialysis catheters to acute emergency situations for a duration of no more than 2 weeks; however, the level of evidence supporting this recommendation is low.
- Guidelines are partially based on studies comparing tunneled hemodialysis catheters with straight non-tunneled catheters used for jugular access.
- Straight non-tunneled catheters used for jugular access have higher infection rates than pre-curved non-tunneled catheters.

**This study adds:**

- In 301 patients, 625 non-tunneled catheter pairs and 53 tunneled catheters were used. The rate of catheter-related blood stream infections (CRBSIs) was identical in both groups (0.48/1000 catheter-days); analyzing possible and confirmed CRBSIs together also showed similar incidence (*P* = .68).
- As a sensitivity analysis, comparison of non-tunneled and tunneled catheters was made in smaller subgroups of patients who had both types of catheters during the study period and those after first CRBSI. Both showed only non-significantly higher CRBSI rate in non-tunneled catheters.

**Potential impact:**

- The prolonged use of non-tunneled, pre-curved hemodialysis catheters, which are easily maintained, is a viable option, especially for patients awaiting construction of arteriovenous fistula, insertion of a peritoneal catheter or kidney transplantation in a reasonable time.

## INTRODUCTION

Patients with end-stage kidney disease can choose from different modalities of renal replacement therapy [1]. Although kidney transplantation is the best option in most cases and peritoneal dialysis is preferred for a subset of patients, the majority initiate treatment with hemodialysis as the first modality of renal replacement therapy [2–5]. Many of these patients remain on hemodialysis due to comorbid conditions or the limited availability of alternative modalities in certain regions of the world [6–9].

For patients undergoing hemodialysis reliable vascular access is essential. An arteriovenous fistula (AVF) or graft is generally the preferred option [10]. If these are not feasible because of severe heart failure, inadequate peripheral vessels or patients’ poor life expectancy, a hemodialysis catheter becomes the only viable alternative [10]. Additionally, a hemodialysis catheter may serve as a practical short- or medium-term solution prior to creation and maturation of an AVF, the insertion of a peritoneal dialysis catheter or a planned kidney transplantation.

Current guidelines strongly recommend almost exclusive use of tunneled cuffed hemodialysis catheters and suggest restricting the use of non-tunneled catheters to acute emergency situations for a duration of no more than 2 weeks. However, the level of evidence supporting this recommendation is low [10] and compliance with this recommendation in the real world is also questionable. The guidelines appear to be largely influenced by a study conducted by Weijmer *et al*., which compared tunneled catheters with straight non-tunneled catheters—commonly used at the time of the study—inserted into internal jugular veins [11]. The difference in infection rates between the two groups was indeed substantial. However, later the same authors published a follow-up study after they had switched to pre-curved non-tunneled catheters for jugular access, demonstrating significantly improved outcomes, with infection rates more comparable to those of tunneled catheters [12]. Moreover, several other studies have also shown similar results of low infection rates for pre-curved non-tunneled jugular catheters, including reports from our center [13–16].

When pre-curved hemodialysis catheters were first introduced at our center, we encountered issues with low blood flow from the arterial lumen of the double-lumen catheters featuring a coaxial lumen design and side holes. Therefore, we adopted the use of two pre-curved single lumen catheters instead, and this approach has been maintained to the present with consistently good results. Given the low infection rates observed with this approach, which were comparable to the published data for tunneled catheters, we have maintained a policy of using two non-tunneled single lumen catheters for all acute patients and most incident chronic hemodialysis patients requiring a catheter (e.g. when AVF has not yet been constructed or matured or is not feasible). According to our center’s policy, we use tunneled catheters only in selected patients who already had at least one catheter-related blood stream infection (CRBSI) or tend to repeatedly remove their non-tunneled catheters due to cognitive impairment.

In this retrospective study, we compared the rate of CRBSIs between non-tunneled and tunneled hemodialysis catheters used for chronic hemodialysis in our center to assess whether our clinical practice—despite deviating from guideline recommendations—remains appropriate.

## MATERIALS AND METHODS

### Study design and data collection

This was a retrospective, single-center study that included chronic hemodialysis patients with non-tunneled or tunneled catheters treated in our tertiary hospital’s hemodialysis center between 1 January 2018 and 1 July 2024. We included patients who were dialyzed on an outpatient basis for at least 1 month and had jugular or subclavian, non-tunneled or tunneled catheters. We analyzed the electronic medical records and extracted data related to vascular access: catheter type, insertion date and insertion site, and all recorded procedures and complications. Patients’ characteristics were also documented at baseline (first catheter insertion). To ensure that all possible catheter-related infections were recorded, we reviewed all hospitalizations during the follow-up period and screened all blood culture results obtained during the study period from the central microbiology database. Follow-up continued until 1 March 2025.

The study was approved by the National Medical Ethics Committee (No. 0120-179/2024-2711-5) and the requirement for informed consent was waived due to the retrospective nature of the study.

### Clinical practice regarding catheters

All catheters were inserted under local anesthesia using aseptic technique and ultrasound guidance and were then sutured to the skin. Non-tunneled catheters were inserted by all nephrologists in the department skilled in central venous catheters insertions, while tunneled catheters were inserted by subgroup of nephrologists specifically skilled in this procedure. Most non-tunneled and tunneled catheters were inserted without the use of fluoroscopy. Two single lumen, pre-curved, 8F (12, 15 or 20 cm) temporary catheters (Medcomp^®^, Harleysville, Pennsylvania, USA) were most often used for jugular access, while straight, 8F (15 or 20 cm) catheters (Medcomp^®^) were mostly used for insertion in subclavian veins. For tunneled catheters, we used dual lumen, 14F (28 cm) or 16F (32 or 36 cm) catheter (Split Cath^®^, Medcomp^®^) or 15.5F (33 cm) catheters (Symetrex^®^, Medcomp^®^). Subclavian veins were used only when jugular access was not feasible. Non-tunneled catheters were removed and subsequently reinserted in cases of suspected or confirmed exit-site infection or suspected or confirmed CRBSI. In most instances, temporary femoral catheters were used during hospitalization until another long-term vascular access could be established. In cases of confirmed CRBSI where AVF creation was not feasible, we often opted to insert a tunneled catheter rather than a non-tunneled one after the infection had resolved. In rare cases where all potential catheter insertion sites were exhausted, catheters were exchanged over the guidewire. Over the wire exchanges were also performed in cases of catheter defect or insufficient flow. Prior to each over-the-wire exchange 1–2 g of cefazolin was administered intravenously.

Tunneled catheters were either removed or salvaged after a confirmed catheter exit-site infection or a confirmed CRBSI. For catheter salvage attempts in case of CRBSI, an antibiotic lock was used together with systemic antibiotics. In cases of insufficient blood flow all newly inserted tunneled catheters were exchanged over a guidewire, with a correction of the tunnel arch. For older tunneled catheters an alteplase (Actilyse, Boehringer Ingelheim International GmbH, Ingelheim am Rhein, Germany) lock or perfusion for 1 h was attempted. In catheters with persistent blood flow issues or a malfunction, a new catheter was inserted with over-the-wire exchange along with the creation of a new tunnel or a new catheter was inserted on a new location.

Catheter care was performed by the dialysis nurse using aseptic technique with the use of a face mask and sterile gloves before or during every hemodialysis session. The catheter exit-site was examined and cleaned with 2% chlorhexidine in 70% alcohol (or pure 70% alcohol in case of skin allergy) and dressing was made with a sterile gauze after 2% mupirocin (Betrion^®^, Pliva Ljubljana, Ljubljana, Slovenia) or a polymyxin B/bacitracin (prepared by the hospital’s pharmacy) ointment was applied to the catheter exit-site. Non-tunneled catheters were locked with either 4% trisodium citrate (prepared by the hospital’s pharmacy) or 30% trisodium citrate (Citra-Lock^®^, Dirinco AG, Wilen bei Wollerau, Switzerland) and tunneled catheters were locked mostly with 4% trisodium citrate or rarely with unfractionated heparin (diluted with 0.9% sodium chloride solution to 2500 U/mL).

### Outcomes

The primary aim of the study was to compare the rates of CRBSIs between tunneled and non-tunneled catheters. The unit of observation was vascular access, defined as a tunneled catheter or two (rarely only one) single-lumen non-tunneled catheters considered collectively as one “vascular access,” based on our clinical practice [15]. CRBSI was defined as systemic infection characterized by positive blood cultures, elevated inflammatory parameters and no alternative identifiable source of infection [17]. Systemic infections with a probable alternative source of infection but where CRBSI could not be excluded were classified as possible catheter-related bloodstream infections (PCRBSIs).

We compared non-tunneled and tunneled catheters in all patients and also in a subgroup of patients who had both tunneled and non-tunneled catheters inserted during the observation period. In our clinical practice tunneled catheters were typically inserted in patients after an episode of CRBSI with a non-tunneled catheter. Such patients may be more susceptible to subsequent CRBSI. Therefore, we also compared infection rates between non-tunneled and tunneled catheters inserted in a subgroup of patients after successful treatment of their first CRBSI.

In-hospital mortality due to CRBSI was also assessed and was defined as death occurring during the hospitalization for a CRBSI or PCRBSI.

### Statistical analysis

Statistical analyses were performed using Statistica 12.0 (StatSoft Europe, Hamburg, Germany). Descriptive statistics were reported as means and standard deviations or percentages, as appropriate. The frequency of infections and interventions was expressed as an incidence rate (number per 1000 access-days) and compared by calculating incidence rate ratio (IRR) with 95% confidence interval (CI) and a chi square test.

Kaplan–Meier survival analysis was used to estimate the time to first CRBSI or CRBSI/PCRBSI. In cases where an AVF was created, catheter observation time was censored at the date of catheter removal. If the date was not available, observation was censored 6 weeks after AVF creation, as this is the typical maturation period before the first cannulation. After arteriovenous graft creation, observation was censored 3 weeks post-construction or on the day of graft construction in cases of early cannulation graft, if the date of catheter removal was not recorded. The difference between the tunneled and non-tunneled catheter groups was assessed using the log-rank test.

Mortality rates were compared using Fisher’s exact test. A *P*-value <.05 was considered significant in all analyses.

## RESULTS

We included data from 301 patients with a mean age of 68 ± 15.5 years; 166 (55.1%) were male and 135 (44.9%) were female. Altogether, 678 catheters or catheter pairs were used in these patients, including 625 non-tunneled and 53 tunneled catheters. Of the total, 446 catheters were newly inserted (among them 38 tunneled), while 232 catheters were exchanged over the wire (among them 15 tunneled). The vast majority of the catheters were inserted in jugular veins, altogether only 9 (1.4%) non-tunneled and 13 (25%) tunneled catheters were inserted in subclavian veins. Among 53 tunneled catheters, 9 (17%) were inserted in patients without a prior catheter, 18 (34%) in patients who had previously experienced a CRBSI with a non-tunneled catheter and 26 (49%) in patients with a non-tunneled catheter, but without a prior CRBSI. Of the 301 included patients, 27 had both tunneled and non-tunneled catheters inserted during the study period and were additionally analyzed as a subgroup.

Total observation period amounted to 110 435 access-days for non-tunneled and 20 625 access-days for tunneled catheters. The median access duration was 107 days for non-tunneled and 213 days for tunneled catheters.

### Catheter-related bloodstream infections

During the observation period, we identified 63 CRBSIs and 22 PCRBSIs, resulting in an overall CRBSI rate of 0.48 per 1000 access-days. The majority of CRBSIs were caused by *Staphylococcus aureus*, accounting for 62% of CRBSI and 32% of PCRBSIs (for detailed information on causative pathogens see Supplementary data, Table S1).

**Figure 1:**
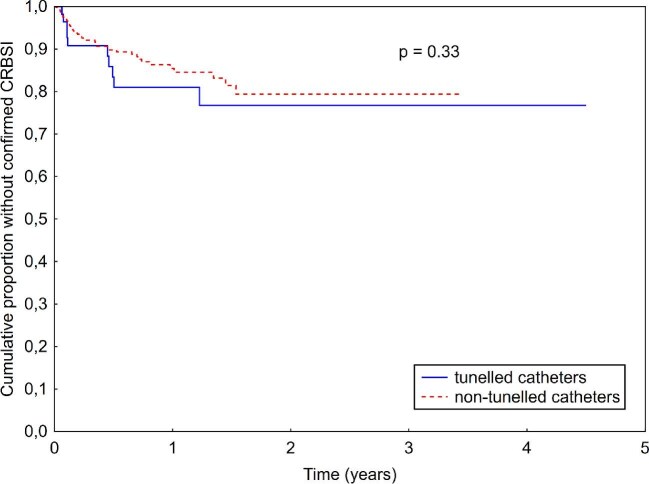
Kaplan–Meier curve comparing time to first confirmed CRBSI between tunneled and non-tunneled catheters.

**Figure 2:**
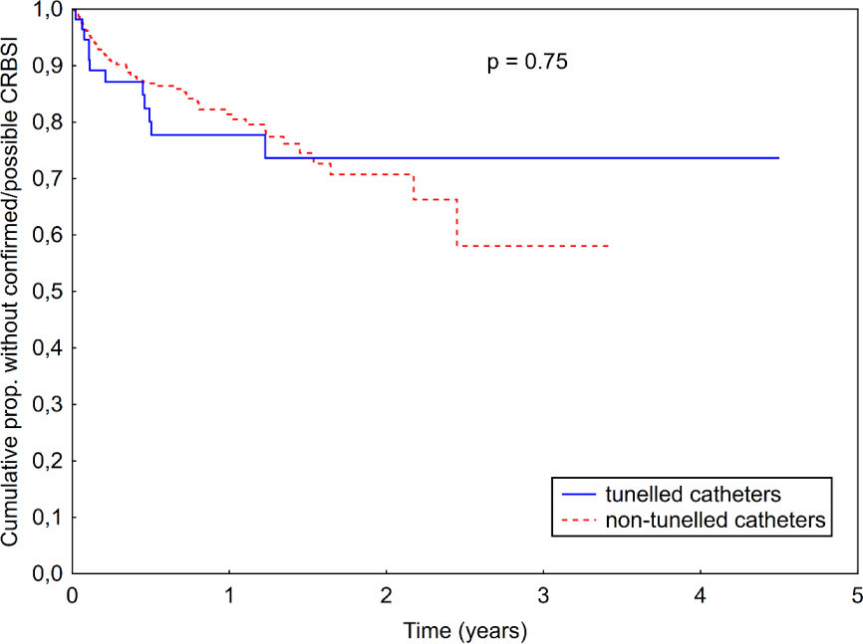
Kaplan–Meier curve comparing confirmed CRBSIs or PCRBSIs between tunneled and non-tunneled catheters.

A total of 53 CRBSIs occurred in non-tunneled catheters and 10 CRBSIs in tunneled catheters, corresponding to an identical incidence rate of 0.48 CRBSIs/1000 access-days in both groups (IRR 0.99, 95% CI 0.5–1.95, *P* = .98, see Table 1). Time to the first CRBSI was also similar between the two groups (log-rank *P* = .33; see Kaplan–Meier curves in Fig. 1). When CRBSIs and PCRBSIs were analyzed together, there were 73 (P)CRBSIs with non-tunneled catheters, compared with 12 (P)CRBSIs with tunneled catheters. As represented in Table 1, this difference was also not statistically significant (IRR 1.14, 95% CI 0.6–2.1, *P* = .68). Kaplan–Meier analysis also showed comparable time to first (P)CRBSI between the groups (log-rank *P* = .75; see Fig. 2).

**Table 1:** Incidence rate for CRBSIs and PCRBSIs in both groups, which are compared by IRR and its 95% CI, for non-tunneled vs tunneled catheters and calculated mortality rates for CRBSI and PCRBSI compared with Fisher’s exact test.

Outcome	All catheters	Non-tunneled catheters	Tunneled catheters	IRR (95% CI)	*P*-value
Observation period (access-days)	131 060	110 435	20 625		
CRBSIs (*N*/1000 access-days)	0.48	0.48	0.48	0.99 (0.5–1.95)	.98
CRBSIs and PCRBSIs (*N*/1000 access-days)	0.56	0.66	0.58	1.14 (0.6–2.1)	.68
Mortality rate for CRBSIs, *n*/*N* (%)	13/63 (20.6)	10/53 (18.9)	3/10 (30)		.42
Mortality rate for CRBSIs and PCRBSIs, *n*/*N* (%)	23/85 (27.1)	18/73 (24.7)	5/12 (41.7)		.29

The mortality rates due to CRBSIs and PCRBSIs were somewhat higher in patients with tunneled catheters; however, the difference was not statistically significant (see Table 1).

### Subgroup of patients with both types of catheters

Due to our clinical practice of frequently placing tunneled catheters in patients who had previously experienced a CRBSI with a non-tunneled catheter, we were able to perform a subanalysis comparing the difference in infection rate between catheter types inserted in the same individuals. A total of 27 patients had both tunneled and non-tunneled catheters inserted during the study period. Their mean age was 69.7 ± 15.6 years, and 30% of them had a CRBSI with the initial non-tunneled catheters.

As expected, the incidence of CRBSIs was higher in this higher-risk subgroup, but still comparable between both catheter types. There were 11 CRBSIs during 11 926 access-days with non-tunneled catheters (0.92/1000 catheter-days) and 8 CRBSIs during 11 911 access-days with tunneled catheters (0.67/1000 catheter-days), yielding an IRR of 1.37 (95% CI 0.55–3.41, *P* = .49). Kaplan–Meier analysis showed no significant difference in time to CRBSI (log-rank *P* = .93; see Supplementary data, Fig. S1). Similar findings were observed for combined CRBSIs and PCRBSIs (1.09/1000 access-days vs 0.84/1000 access-days, IRR 1.3, 95% CI 0.57–2.96, *P* = .47), log-rank *P* = .84 (see Kaplan–Meier curve in Supplementary data, Fig. S2).

### Subgroup of patients after first CRBSI

We also conducted a subanalysis of patients who were successfully treated for their first CRBSI and subsequently had a new, non-tunneled or tunneled catheter inserted. A total of 36 such patients were identified with a mean age of 68.5 ± 13.9 years. Of these, 11 had a tunneled catheter inserted following their initial CRBSI, while the remaining 25 had a non-tunneled catheters reinserted for the second time.

There were seven CRBSIs among non-tunneled catheters during 7397 access-days and three CRBSIs with tunneled catheters during 6396 access-days, which translates to CRBSI rates of 0.95 and 0.47/1000 access-days. The difference was still not statistically significant (see Table 2). There was also no difference between the two groups in the time to CRBSI (log-rank *P* = .60, see Supplementary data, Fig. S3). When comparing CRBSIs and PCRBSIs together, there were 10 (P)CRBSIs with non-tunneled catheters and 4 (P)CRBSIs with tunneled catheters. Again, this difference was not statistically significant (see Table 2). Time to first (P)CRBSI was also similar between the groups (log-rank *P* = .59; see Supplementary data, Fig. S4), though the small sample size limits the statistical power of these comparisons.

**Table 2:** Incidence rate for CRBSI and PCRBSI in a subgroup of patients after their first CRBSI; comparison by IRR and its 95% CI for non-tunneled vs tunneled catheters.

Outcome	All catheters	Non-tunneled catheters	Tunneled catheters	IRR (95% CI)	*P*-value
Observation period (access-days)	13789	7393	6369		
CRBSI (*N*/1000 access-days)	0.73	0.95	0.47	2.01 (0.52–7.72)	.47
CRBSI and PCRBSI (*N*/1000 access-days)	1.02	1.35	0.63	2.16 (0.68–6.9)	.47

## DISCUSSION

In this retrospective study, we observed a comparable incidence of CRBSIs between non-tunneled and tunneled hemodialysis catheters in our center. These findings support the adequacy of our current clinical approach, which diverges from existing guidelines [10], as we routinely use non-tunneled dialysis catheters for extended periods and reserve tunneled catheters primarily for patients with complications.

The KDOQI Clinical Practice Guideline for Vascular Access [10] is partially based on the study by Weijmer *et al*., which reported a striking difference in infection rates between tunneled and non-tunneled catheters, becoming significant after only 14 days of use [11]. In that study, the infection rate for tunneled catheters was 2.9/1000 catheter-days, while the rate for non-tunneled catheters was approximately four times higher, at 12.8/1000 catheter-days. It is important to note that, at that time, non-tunneled catheters used for jugular access were straight rather than pre-curved, as is now the standard.

In contrast, our current study demonstrated much lower infection rate of 0.58/1000 catheter-days for tunneled and 0.66/1000 access-days for non-tunneled pre-curved catheters (looking at CRBSIs and PCRBSIs together). These findings suggest a general decline in CRBSI rates, which might be partially attributed to the use of pre-curved jugular temporary catheters, which were shown to reduce infection rates [5]. Notably, in a follow-up study conducted after switching to pre-curved non-tunneled jugular catheters, Weijmer *et al.* reported no infectious complications over 2101 catheter-days and concluded that their use may be safe for up to 3 months [12].

We have previously published our experience with using pre-curved, non-tunneled jugular hemodialysis catheters as permanent vascular access in patients without a possibility for AVF placement. In those reports, infection rates were low, ranging between 0.2 and 0.4/1000 catheter-days [14–16]. In the present study, we report on the largest cohort from our center to date. Although the infection rates are higher than in our previous reports, they remain comparable to recent reports from other centers. For instance, a retrospective study by Van Oevelen *et al*. reported 0.66 systemic infections/1000 catheter-days with tunneled catheters and 0.84 infections/1000 catheter-days with pre-curved jugular non-tunneled catheters [13]. A retrospective study from Spain found a slightly lower CRBSI rate for tunneled catheters at 0.4 per 1000 catheter-days; however, that analysis included only confirmed CRBSIs [18], whereas our study also included possible CRBSIs.

In general, one would expect a lower infection risk for tunneled catheters, as the subcutaneous tunnel should prevent the spread of infection from the exit-site to the circulation. While the previously mentioned study by Van Oeleven *et al*. [13] found no difference in infection rates between tunneled and non-tunneled catheters, a more recent study by Lima *et al*. [19] did report lower infection rate associated with tunneled catheters. However, their analysis included all catheter insertion sites, including femoral sites, which are known to carry a higher risk of infection [11]. Lawrence *et al*. also reported a difference in CRBSI rates between tunneled and non-tunneled catheters (0.53 vs 1.25/1000 catheter-days), although it did not reach statistical significance (*P* = .1) [20].

In our study, we observed no difference in confirmed CRBSI rates between tunneled and non-tunneled catheters. There was a non-significant, 14% higher rate of (P)CRBSI and a 37% higher CRBSI rate in a small subgroup of patients who had both catheter types. Among high-risk patients who were successfully treated for the first CRBSI—who are likely more susceptible to reinfection—we found twice higher risk of CRBSI with non-tunneled catheters, although the difference did not reach statistical significance, probably due to low number of CRBSIs in both groups and low number of tunneled catheters, which translated to wide CIs. Nevertheless, this comparison still suggests that tunneled catheters may offer some protection in patients at high risk for infection, e.g. after a CRBSI. Contrary to what is stated in the current guidelines, the Kaplan–Meier curves from our study (Supplementary data, Fig. S4) suggest that this potential reduction in CRBSI rates with tunneled catheters becomes apparent only after approximately 6–12 months of use. None of these findings was statistically significant and the differences observed in our study were also smaller than those reported in most of the earlier literature. Additionally, it is also possible that we overestimated CRBSI rates in non-tunneled catheters, since many non-tunneled catheters were in place while AVFs were maturing and in cases where catheter removal dates were missing, we assumed removal occurred 6  weeks after AVF creation. Many patients may have retained their catheters for longer, leading to an underestimation of catheter-days and an overestimation of infection rates.

Our study has several limitations. As noted, not all dates of catheter removal were precisely documented, which could lead to a slight overestimation of CRBSIs in non-tunneled catheters. Since our center’s policy was mostly to insert tunneled catheters in patients who already had one CRBSI with non-tunneled catheter and those patients could be more prone to CRBSIs, this could result in an indication bias and slightly worse CRBSI results for tunneled catheters. We attempted to mitigate this with a separate subanalysis of patients who had previously experienced a CRBSI. Additionally, the total number of tunneled catheters in our study was low, which limits the statistical power of some comparisons and may be the cause why the differences in the incidence of CRBSIs in both subanalyses of patients, which were more prone to CRBSIs, did not reach the statistical significance. Another limitation is the difference in catheter locking solution (a minority of tunneled catheters were locked with unfractionated heparin, whereas all non-tunneled catheters were locked with either 4% or 30% trisodium citrate). Trisodium citrate has been reported to reduce infection risk compared with heparin in some studies [21–23] though not in all [24–26]. According to a recent meta-analysis [27] trisodium citrate may be the most effective when used in combination with antibiotics or at lower concentrations. In our study, however, trisodium citrate was also used in higher concentrations for non-tunneled catheters. Additionally, we occasionally used alteplase in tunneled catheters with suboptimal function which has also been reported to decrease CRBSI risk as well [28], and may therefore have provided some protective benefit for tunneled catheters.

In our dialysis center, AVFs are created by skilled nephrologists, and waiting times for AVF construction are typically <1 month. An environment where every nephrologist can insert a non-tunneled jugular catheter when needed and AVF can be created promptly by a trained nephrologist works well, and the results of this study do not indicate a need to change our practice. Even though we have not found significant differences in infection rates between tunneled and non-tunneled catheters, we cannot recommend the widespread adoption of our policy—favoring non-tunneled catheters as default vascular access in chronic hemodialysis patients—for all dialysis centers. Our study focused only on the hard outcome of CRBSIs and did not assess catheter function or patients’ quality of life. Although the difference in CRBSI rates between groups was not statistically significant, the trend of higher CRBI rate for non-tunneled catheters appeared among patients with higher predisposition to CRBSI. Nevertheless, we think that these data should encourage us to perform a randomized clinical trial to compare both types of catheters. Moreover, centers experiencing high infection rates with non-tunneled catheters should be encouraged to reassess their catheter management protocols.

In our view, tunneled catheters remain the preferred option for permanent vascular access in patients who are more prone to CRBSIs or in whom such infection would be of particular concern—for example, in those with implanted artificial materials. They are also preferable for patients with cognitive impairment, where accidental catheter pull-outs are a concern. However, the current recommendations discouraging the short- or medium-term use of non-tunneled catheters before an early AVF creation, insertion of a catheter for peritoneal dialysis or kidney transplantation should be called into question. The long-term use of non-tunneled catheters in patients without a possibility for an AVF or graft creation should be further studied in prospective randomized trials.

## CONCLUSION

Our retrospective study comparing tunneled and non-tunneled, but pre-curved catheters in chronic hemodialysis patients found no significant difference in the incidence of catheter-related bloodstream infections with prolonged use, but these results need to be confirmed in prospective studies. We conclude that the use of non-tunneled, pre-curved hemodialysis catheters is a viable option for patients awaiting construction of an arteriovenous fistula, insertion of a catheter for peritoneal dialysis or kidney transplantation in a reasonable time. However, tunneled catheters should remain the preferred option for permanent vascular access, especially in patients with an increased risk of CRBSI, implanted artificial materials or in those with cognitive impairment where accidental pull-outs are a concern.

## Supplementary Material

sfaf392_Supplemental_File

## Data Availability

The raw data supporting the conclusions of this article will be made available by the authors without undue reservation.
