# Supplementary material for: Comparable rates of catheter-related bloodstream infections between non-tunneled and tunneled hemodialysis catheters: a retrospective single-center study
Source: Clin Kidney J. 2025 Dec 16;19(1):sfaf392. doi: 10.1093/ckj/sfaf392 (PMC12780762; doi:10.1093/ckj/sfaf392)
Supplement: sfaf392_Supplemental_File [file sfaf392_supplemental_file.docx]

**Supplementary table 1.** Total number and calculated frequency of the type of isolated pathogens in catheter related bacteremia and possible catheter related bacteremia. S. - Staphylococcus.

| **Isolated pathogens in catheter related bacteremias** | No. | (%) |
| --- | --- | --- |
| Staphyloccocus aureus | 39 | 62% |
| Methicillin-resistant Staphylococcus aureus (MRSA) | 2 | 3% |
| Staphylococcus epidermidis | 4 | 6% |
| Staphylococcus epidermidis and Escherichia coli | 2 | 3% |
| Enterobacter cloace | 2 | 3% |
| Staphylococcus aureus and Staphylococcus epidermidis | 1 | 2% |
| Staphylococcus epidermidis and Pseudomonas aeroginosa | 1 | 2% |
| Staphylococcus epidermidis and Staphylococcus warneri | 1 | 2% |
| Staphylococcus hominis | 1 | 2% |
| Staphylococcus agalactiae | 1 | 2% |
| S. aureus, S. epidermidis, S. pettenkoferi and S. lugdunensis | 1 | 2% |
| Pseudomonas aeroginosa | 1 | 2% |
| Streptococcus salivarius | 1 | 2% |
| Streptococcus constellatus ssp. constellatus | 1 | 2% |
| Citrobacter koseri | 1 | 2% |
| Proteus mirabilis and Candida albicans | 1 | 2% |
| Enteroccocos faecium | 1 | 2% |
| Proteus mirabilis | 1 | 2% |
| S. aureus, Staphylococcus lugdunensis, and Klebsiella oxcxtoca | 1 | 2% |
| **Isolated pathogens in possible catheter related bacteremias** |  |  |
| Staphyloccocus aureus | 7 | 32% |
| Methicillin-resistant Staphylococcus aureus (MRSA) | 3 | 14% |
| Staphylococcus epidermidis | 2 | 9% |
| Proteus mirabilis | 2 | 9% |
| Pseudomonas aeroginosa | 2 | 9% |
| Pseudomonas aeroginosa and Morganella morganii | 1 | 5% |
| Rhodococcus equi | 1 | 5% |
| Streptococcus constellatus ssp. constellatus | 1 | 5% |
| Streptococcus dysgalactiae subsp. equisimilis | 1 | 5% |
| Unknown | 2 | 9% |


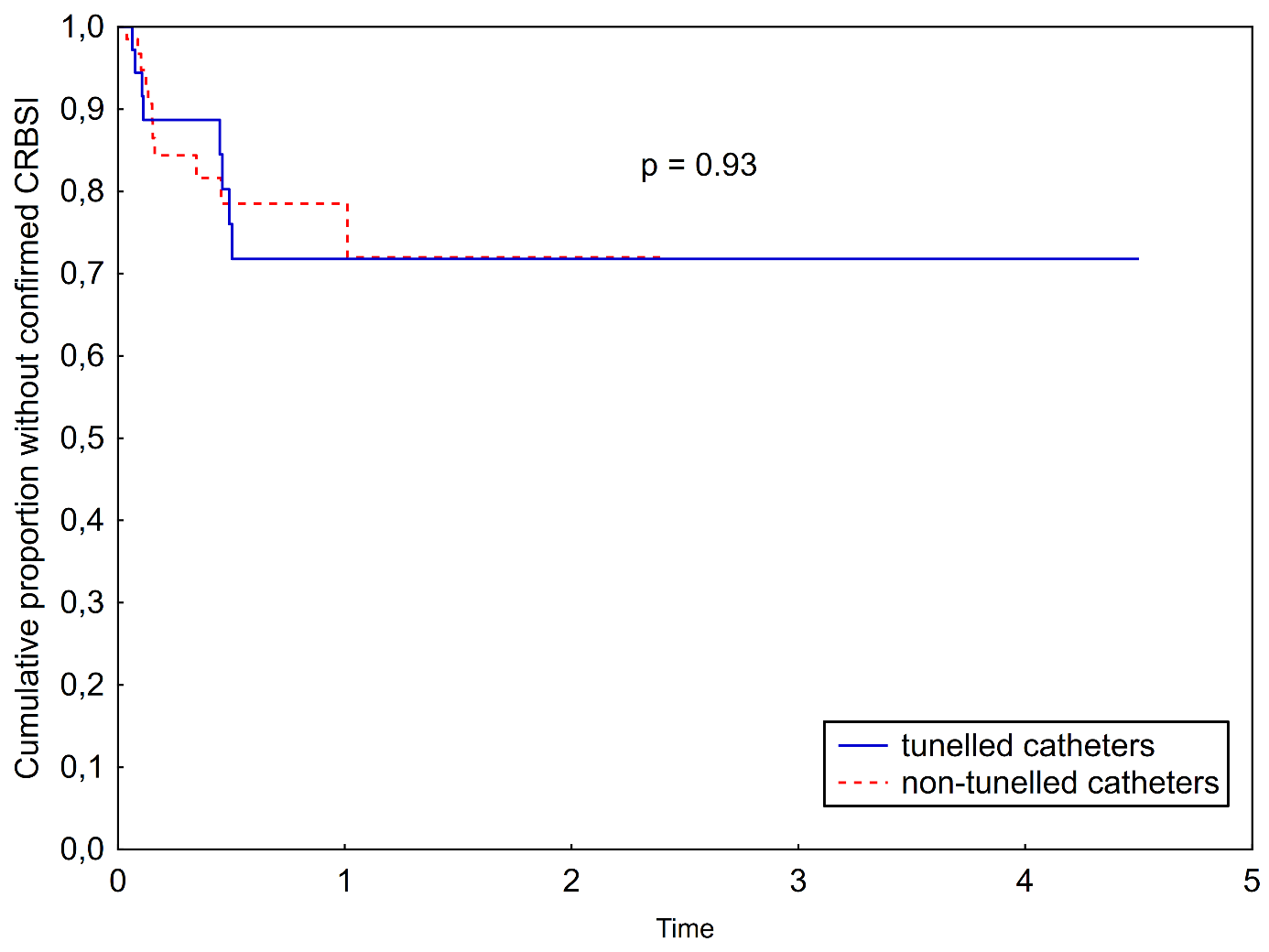


**Supplementary Figure 1.** Kaplan-Meier curve comparing confirmed catheter-related bloodstream infections between tunneled and non-tunneled catheters for patients who were dialyzed by both types of catheters during the observation period.


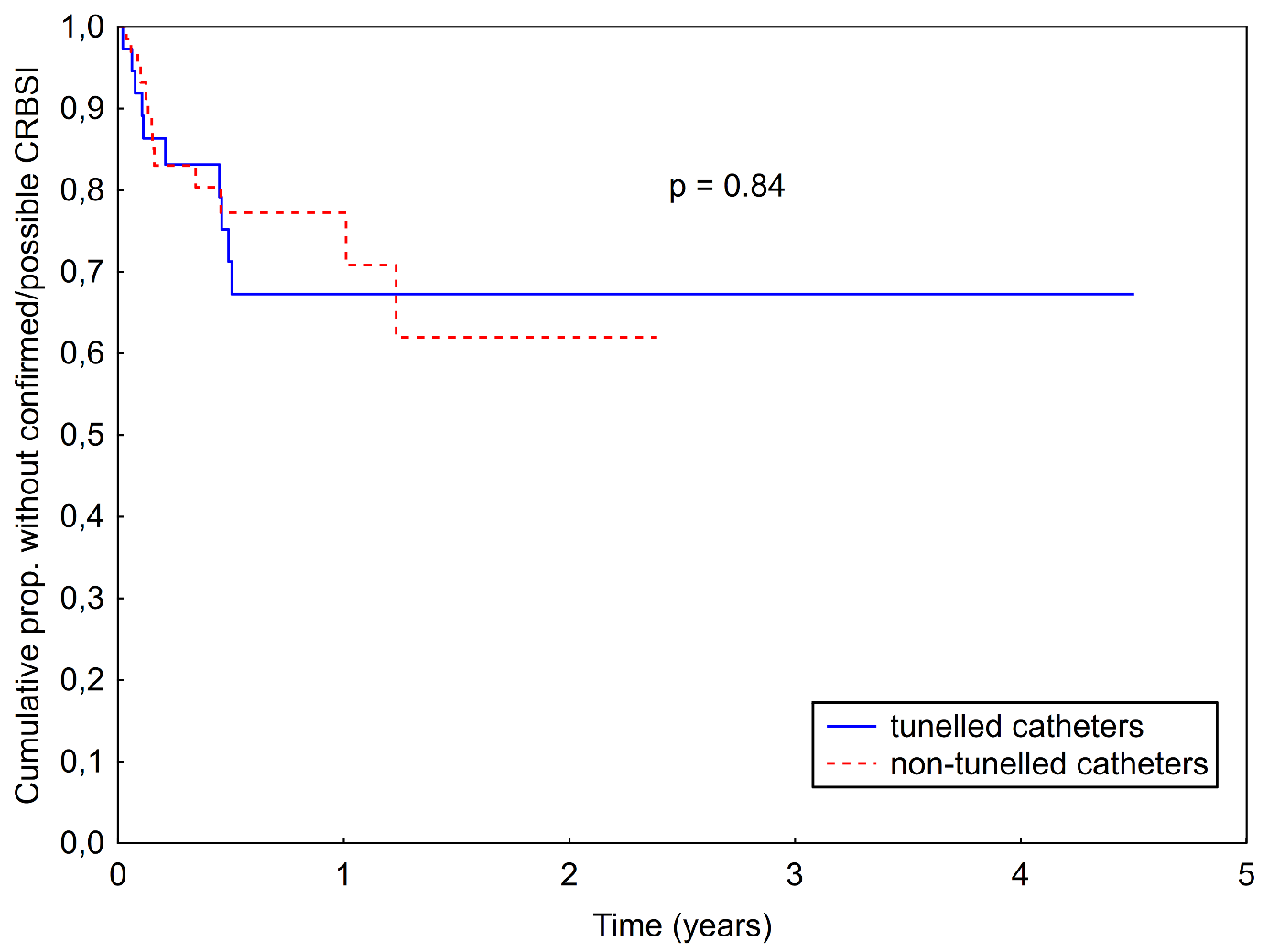


**Supplementary Figure 2.** Kaplan-Meier curve comparing confirmed or possible catheter-related blood-stream infections between tunneled and non-tunneled catheters for patients who were dialyzed by both types of catheters during the observation period.


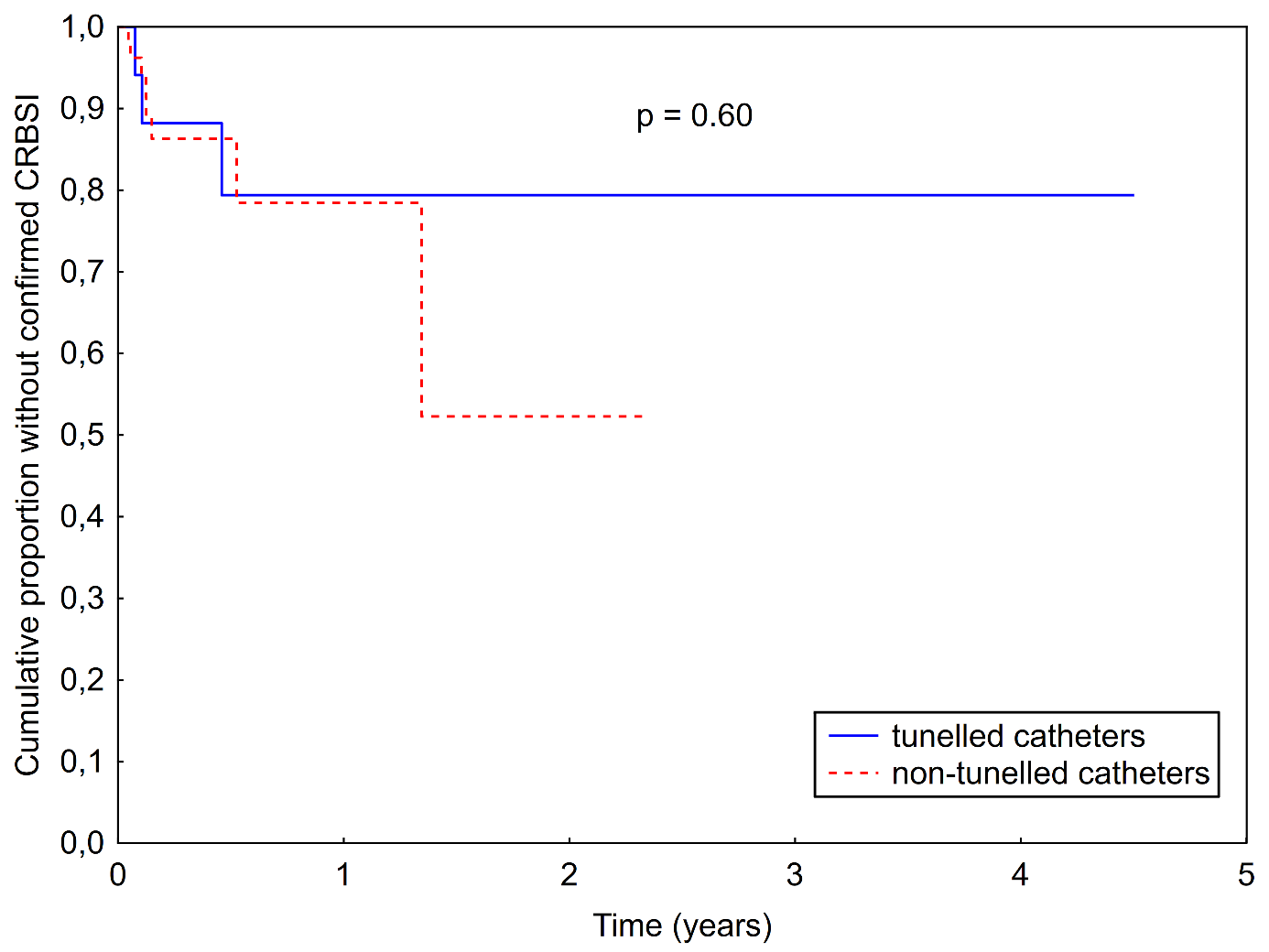


**Supplementary Figure 3.** Kaplan-Meier curve comparing confirmed catheter-related blood-stream infections between tunneled and non-tunneled catheters for patients who already had one (p)CRBSI during the observation period.


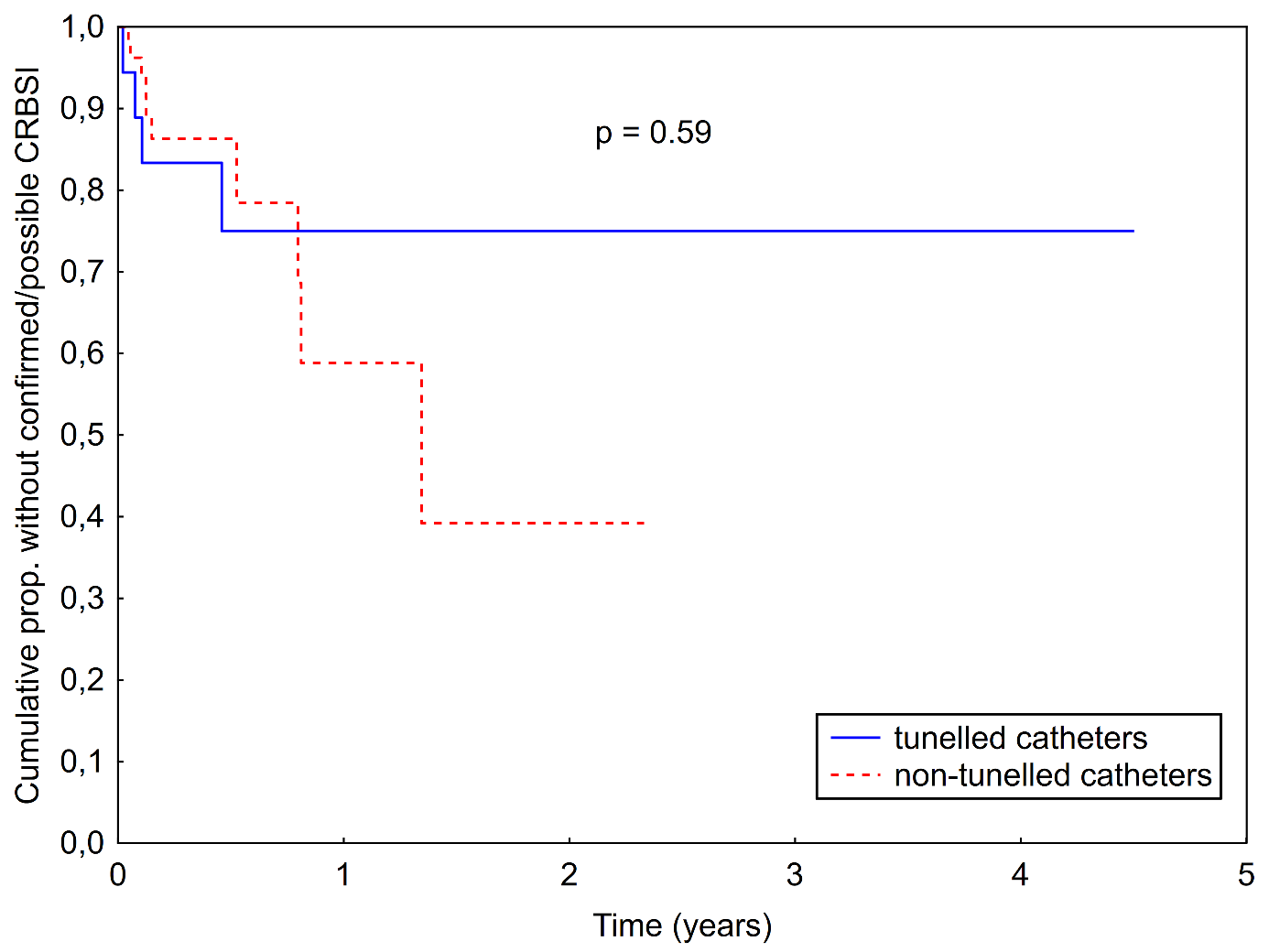


**Supplementary Figure 4.** Kaplan-Meier curve comparing confirmed or possible catheter-related blood-stream infections between tunneled and non-tunneled catheters for patients who already had one (p)CRBSI during the observation period.
